# Sarcoid Pleural Effusion Presenting With Pachypleuritis: A Rare Manifestation in a Young Adult

**DOI:** 10.7759/cureus.95800

**Published:** 2025-10-31

**Authors:** Leticia Balanco, Vânia Almeida, Pedro G Ferreira

**Affiliations:** 1 Pulmonology, Unidade Local de Saúde de Coimbra, Coimbra, PRT; 2 Pathology, Unidade Local de Saúde de Coimbra, Coimbra, PRT

**Keywords:** corticosteroids, non-caseating granulomas, pachypleuritis, pleural biopsy, pleural effusion, sarcoid-associated pleural effusion, sarcoidosis

## Abstract

Sarcoidosis is a systemic granulomatous disease of unknown etiology that predominantly involves the lungs and mediastinal lymph nodes. Although other organs may be affected, sarcoid-associated pleural effusion (SAPE) is relatively rare and often poses a diagnostic challenge. We report a case of a 24-year-old male who presented with pleural effusion (PE) and fever. Thoracentesis revealed an exudative PE with lymphocytosis. Microbiological studies were negative. He was empirically treated with amoxicillin/clavulanic acid and azithromycin for community-acquired pneumonia. Chest computed tomography showed nodular pleural thickening and a persistent small-volume PE. The patient underwent pleural decortication, and excisional biopsy of a lingular lymph node confirmed sarcoidosis. One year later, he developed functional impairment, and positron emission tomography-computed tomography revealed several hypermetabolic lesions, prompting corticosteroid therapy with a good response. At two years, lung function had normalized, residual pleural thickening remained stable, and treatment was withdrawn. Despite its low incidence, SAPE should be considered in the differential diagnosis of pleural effusion, particularly in young patients without comorbidities and with a high pleural fluid lymphocyte count. Exclusion of tuberculosis is fundamental for an accurate diagnosis, and in cases of active pleural disease, systemic treatment is essential to prevent pachypleuritis.

## Introduction

Sarcoidosis is a multisystem granulomatous disease of unknown etiology, most commonly affecting the lungs and intrathoracic lymph nodes, and typically occurring in adults under 50 years of age, with an estimated prevalence ranging from two to 160 cases per 100,000 individuals [1]. Although other organs may be involved, sarcoidosis-associated pleural effusion (SAPE) is a relatively uncommon manifestation of the disease, and no standardized diagnostic criteria have been established for this entity [2]. SAPE is caused by active granulomatous inflammation of the pleura and usually develops within the first year after diagnosis [3]. The diagnostic process requires the exclusion of alternative causes of pleural effusion, which can be established clinically or definitively confirmed through a pleural biopsy demonstrating non-caseating granulomas. In fewer than half of the published SAPE reports, a pleural biopsy was performed to confirm the diagnosis [2]. Glucocorticoids are the cornerstone of treatment, generally allowing a good clinical response and low rates of recurrence [4]. Here, we describe a case of sarcoidosis presenting with pleural effusion and pachypleuritis. Surgical biopsy demonstrated non-caseating granulomas, establishing the diagnosis of SAPE.

## Case presentation

**Figure 1:**
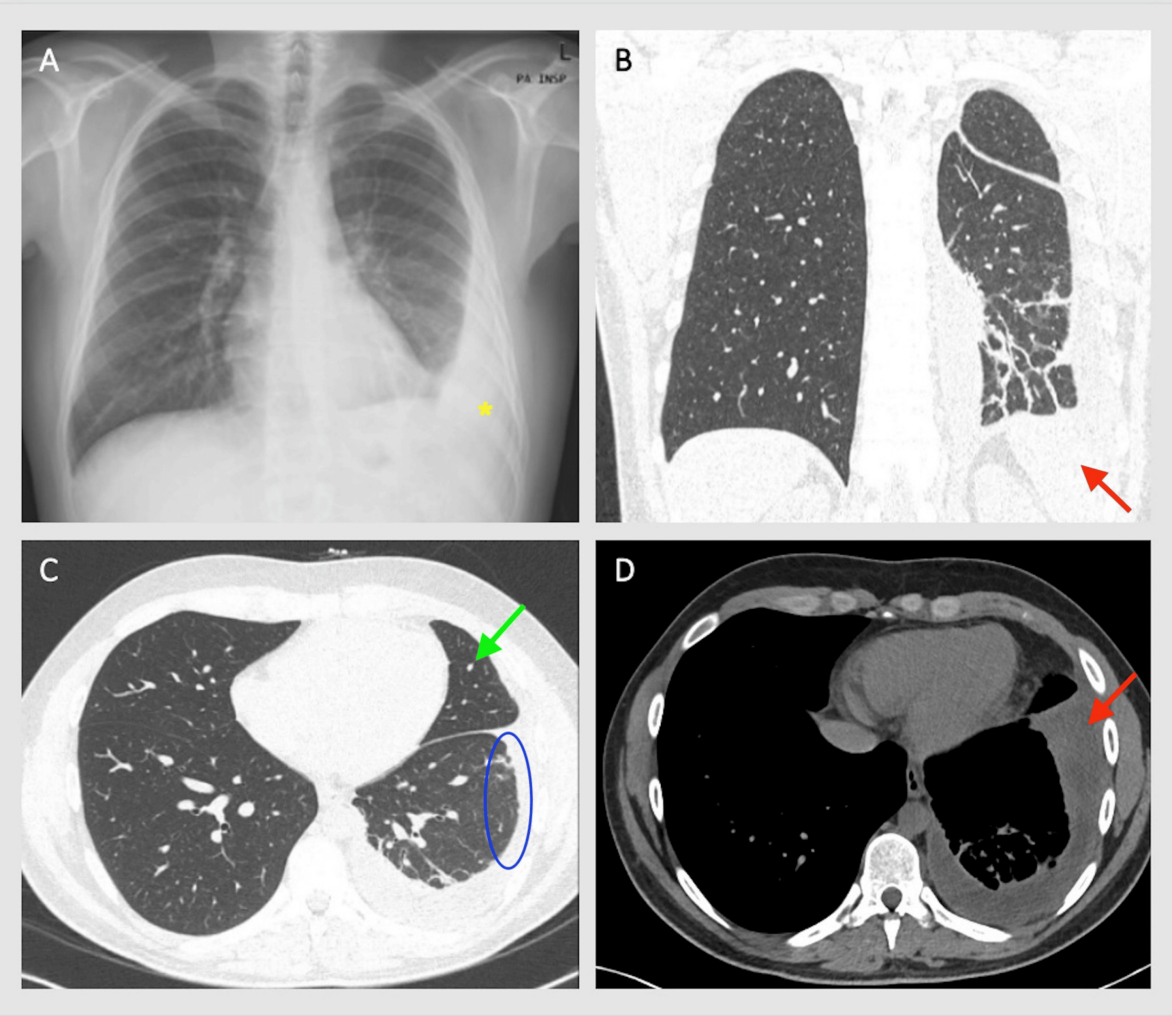
Chest radiography and chest HRCT images before initiation of glucocorticoid therapy. (A) Chest radiography showing left pleural effusion (yellow asterisk). (B) HRCT on the coronal plane reveals small-volume, organized, left-sided pleural effusion (red arrow). (C) HRCT on the axial plane demonstrating nodular pleural thickening (blue circle) and a 4 mm lingular nodule (green arrow). (D) HRCT on the axial plane showing organized left-sided pleural effusion (red arrow). HRCT: high-resolution computed tomography

A 24-year-old male was admitted to the hospital with dyspnea, fever, and left pleural effusion. He was an ex-smoker of seven pack-year. The patient had been well until four weeks prior, when dry cough and dyspnea emerged. Four days before hospital admission, cough and dyspnea worsened, and fever developed. Physical examination was noticeable for low-grade fever, tachycardia, and muffled breath sounds in the left lower lung field. Chest radiography revealed a moderate volume pleural effusion (PE), and chest computed tomography (CT) showed linear subpleural parenchymal bands in the left lower lung, nodular pleural thickening, small volume organized PE, and a 4 mm nodule at the lingula (Figures 1A-1D).

Thoracentesis revealed an exudative PE according to Light’s criteria, with pleural fluid protein 5.0 g/dL and lactate dehydrogenase (LDH) 891 U/L, compared with serum protein 6.6 g/dL (normal: 6.6-8.3 g/dL) and LDH 177 U/L (normal: <248 U/L); the fluid-to-serum protein ratio was 0.77 (>0.5) and the fluid-to-serum LDH ratio was 5.0 (>0.6). The pleural fluid showed marked lymphocytosis (98%; normal: <50%) and elevated adenosine deaminase (ADA) levels (138 U/L; normal: <40 U/L). Mycobacteriological examination of the pleural fluid was negative. Microbiological workup of the sputum and bronchoalveolar lavage fluid was also negative. Autoimmune screen was negative, namely antibody anti-dsDNA, anti-cyclic citrullinated peptide, antinuclear antibody, and anti-neutrophil cytoplasmic antibody. HIV and hepatitis C virus (HCV) serologies were negative, and serologic testing for hepatitis B virus (HBV) was consistent with his post-vaccination status. The patient was treated empirically for community-acquired pneumonia/parapneumonic effusion. He was discharged home clinically stable and referred to thoracic surgery. Given the presence of organized pleural effusion and nodular pleural thickening, the patient was referred for pleural decortication to obtain diagnostic tissue, relieve pleural restriction, and prevent progression to trapped lung.

**Figure 2:**
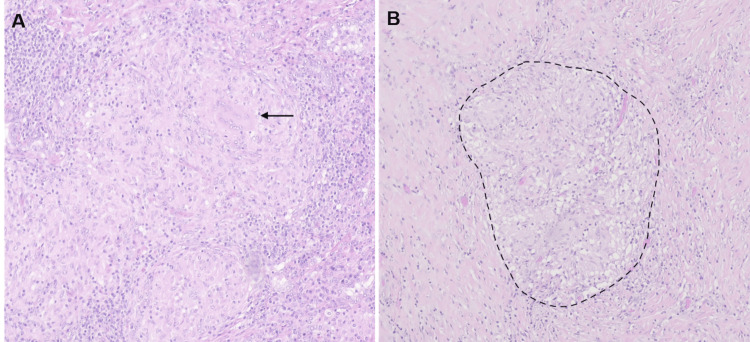
Histopathology of VATS lung and pleural biopsies. (A) Lung parenchyma showing multiple non-necrotizing epithelioid granulomas with a central multinucleated giant cell (arrow) (H&E: ×100). (B) Pleura with extensive hyalinization and well-formed non-necrotizing granulomas (dashed line), morphologically similar to those observed in the lung (H&E: ×40). VATS: video-assisted thoracoscopic surgery

One month later, the patient underwent pleural decortication and excisional biopsy of the lingular node. Histopathological analysis of the lingular node revealed extensive lung involvement by non-necrotizing epithelioid granulomas, characterized by multinucleated giant cells and lymphocytes on the periphery; morphologically similar, numerous non-caseating granulomas were also identified on the pleura (Figures 2A, 2B). Histochemical studies, namely those using Ziehl-Nielsen, Grocott, and periodic acid-Schiff (PAS) stains, revealed the absence of specific microorganisms. Therefore, the diagnosis of sarcoidosis and SAPE was established.

**Figure 3:**
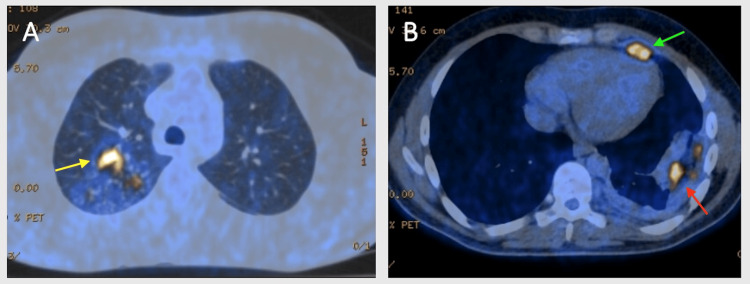
¹⁸F-FDG-PET/CT before glucocorticoid treatment. (A) Hypermetabolic nodules in the right upper lobe (yellow arrow). (B) Hypermetabolic lingular mass (green arrow) and nodular left-sided pachypleuritis (red arrow). ¹⁸F-FDG-PET/CT: fluorodeoxyglucose positron emission tomography/computed tomography

During a follow-up visit one year after surgery, the patient remained asymptomatic; however, his blood tests revealed an elevated serum angiotensin-converting enzyme level (62 U/L, normal range: 8-52 U/L). Consequently, a fluorodeoxyglucose positron emission tomography/computed tomography (¹⁸F-FDG PET/CT) was performed to evaluate potential subclinical disease activity, which revealed hypermetabolic nodules in the upper right lobe, a lingular mass, and left nodular hypermetabolic pachypleuritis (Figures 3A, 3B). Pulmonary function tests (PFTs) at this stage demonstrated a restrictive ventilatory defect with total lung capacity (TLC) 74% predicted, forced vital capacity (FVC) 70% predicted, forced expiratory volume in one second (FEV₁) 72% predicted, FEV₁/FVC ratio: 0.83, and diffusing capacity of the lungs for carbon monoxide (DLCO) 72% predicted. Given the evidence of active pleural disease and this functional limitation, the patient was started on systemic prednisolone 40 mg/day for four weeks and then slowly tapered to a maintenance dose of 5 mg/day.

**Figure 4:**
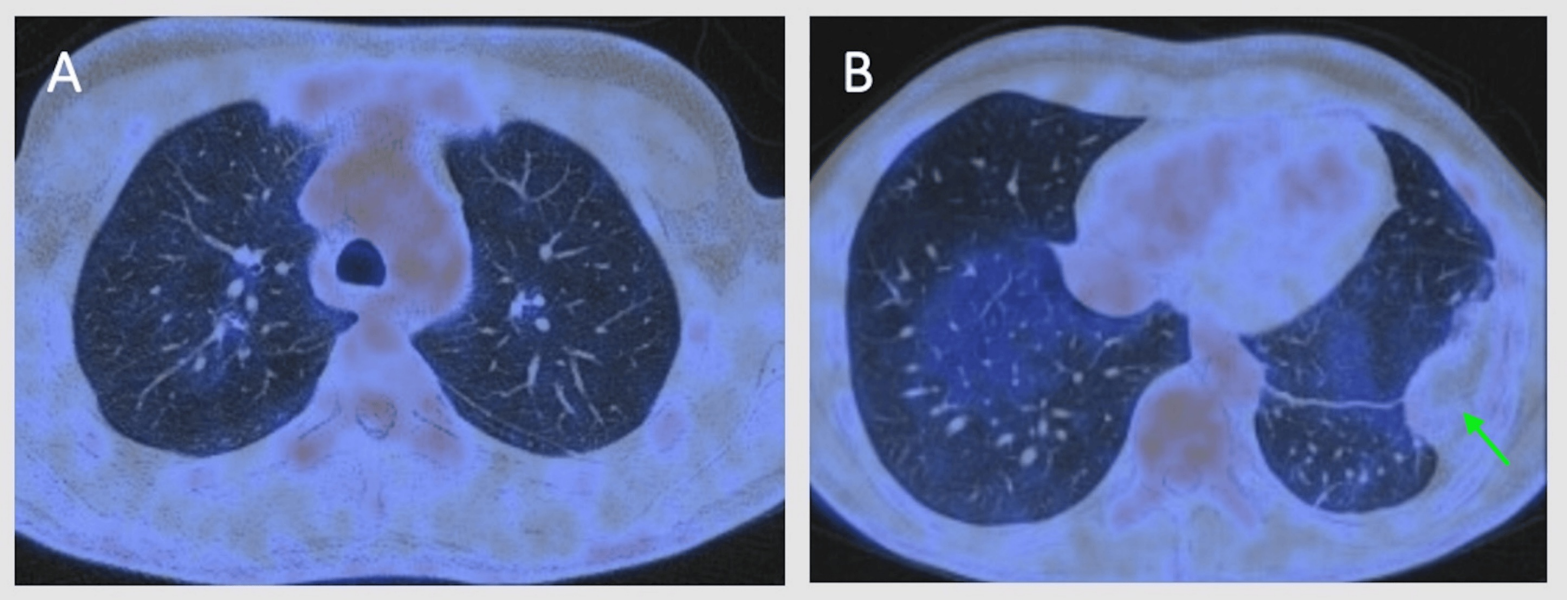
¹⁸F-FDG-PET/CT after glucocorticoid treatment. (A) Resolution of hypermetabolic nodules in the right upper lobe. (B) Sequelae of left-sided pachypleuritis (green arrow) without hypermetabolic activity. ¹⁸F-FDG-PET/CT: fluorodeoxyglucose positron emission tomography/computed tomography

PET-CT reevaluation at 24 months revealed residual pleural thickening, predominantly left-sided of reduced extent and without significant tracer uptake; no evidence of active disease was detected elsewhere (Figures 4A, 4B).

The patient was asymptomatic, and there was also improvement in PFTs (TLC 85% predicted, FVC 75% predicted, FEV₁ 76% predicted, FEV₁/FVC ratio 0.84, and DLCO 100% predicted). Glucocorticoid therapy was gradually tapered to complete withdrawal.

## Discussion

Sarcoidosis is a multisystem granulomatous disease of unknown etiology, most commonly involving the lungs and intrathoracic lymph nodes. Pleural involvement, however, is uncommon and may present as pleural effusion and pleural thickening [1,5]. In this patient, sarcoidosis manifested initially with pleural effusion and later with pleural thickening and parenchymal nodules, a presentation compatible with SAPE, a rare entity that can mimic tuberculosis, lymphoma, or autoimmune pleuritis.

The differential diagnosis of lymphocytic exudative pleural effusion with high ADA is challenging. Tuberculosis is the leading cause worldwide, and ADA levels >40 U/L strongly suggest tuberculous pleurisy [6]. In our patient, ADA reached 138 U/L, initially favoring this diagnosis. However, repeated negative microbiological studies and the histopathological finding of non-caseating granulomas without microorganisms excluded mycobacterial disease. Sarcoidosis must remain a differential diagnosis when ADA is markedly elevated, but direct microbiological evidence of tuberculosis is lacking.

Histological confirmation remains the cornerstone for diagnosis, particularly in atypical or extrapulmonary presentations. In the present case, both pleura and lung biopsy revealed non-necrotizing granulomas without organisms, fulfilling the diagnostic criteria for sarcoidosis. The excisional biopsy of the lingual nodule was decisive, as it ruled out malignancy and confirmed the granulomatous nature of the disease.

The role of PET-CT in sarcoidosis is increasingly recognized. It allows for whole-body assessment, detection of occult organ involvement, and monitoring of disease activity. In our patient, PET-CT revealed pleural and pulmonary hypermetabolic lesions, guiding treatment decisions. After corticosteroid therapy, a subsequent PET-CT scan showed significant improvement, with residual, metabolically inactive pleural thickening, consistent with a good treatment response.

Pulmonary function tests (PFTs) are useful for both baseline evaluation and monitoring treatment. In the present case, restrictive impairment and reduced diffusing capacity of the lungs for carbon monoxide (DLCO) reflected functional limitation associated with pleural and parenchymal disease. Notably, following corticosteroid therapy, the patient demonstrated recovery of lung volumes and normalization of gas exchange capacity, supporting the benefit of systemic treatment in SAPE with functional compromise.

Corticosteroids remain the mainstay of therapy for symptomatic or functionally significant sarcoidosis. Evidence regarding the treatment of SAPE is mainly derived from case reports and small series. While asymptomatic pleural involvement may be self-limiting, the presence of dyspnea, persistent pleural thickening, and restrictive physiology justifies systemic treatment. The functional and imaging improvements observed align with previous reports that SAPE generally carries a favorable prognosis when treated appropriately [4].

## Conclusions

Definitive diagnosis of SAPE is often challenging. This case highlights several key aspects as follows: sarcoidosis should be considered in the differential diagnosis of exudative lymphocytic pleural effusion with elevated ADA, particularly when microbiological tests are negative. Histological confirmation is crucial to distinguish sarcoidosis from tuberculosis or malignancy, while PET-CT proves valuable in assessing both disease extent and activity. Moreover, systemic corticosteroids may lead to symptom resolution and functional recovery in SAPE. Given the rarity of pleural involvement in sarcoidosis, further studies are needed to refine diagnostic algorithms and establish evidence-based treatment guidelines for this manifestation of the disease.
